# An Evaluation of a Mobile App for Chronic Low Back Pain Management: Prospective Pilot Study

**DOI:** 10.2196/40869

**Published:** 2022-10-13

**Authors:** Jonathan D Browne, Michael Vaninetti, David Giard, Konstantinos Kostas, Ankur Dave

**Affiliations:** 1 School of Medicine California University of Science and Medicine Colton, CA United States; 2 Center for Pain Medicine University of California San Diego La Jolla, CA United States; 3 College of Psychology California Northstate University Rancho Cordova, CA United States; 4 Ascension Illinois Alexian Brothers Medical Center Elk Grove Village, IL United States

**Keywords:** back pain, chronic pain, mobile, app, multidisciplinary care, biopsychosocial, self-management, mHealth, mobile health, mobile app

## Abstract

**Background:**

Chronic low back pain is challenging to manage due to multidisciplinary considerations. It has substantial socioeconomic impacts and cannot be simply treated with pharmacotherapy, nonsurgical intervention, or spine surgery. Medical consensus recommends optimizing conservative self-management therapies (eg, home exercise, wellness strategies, yoga, etc) as first-line treatment options for chronic low back pain. However, access to these modalities is often limited and secondary to cost, convenience, and ease of use. Mobile health apps have emerged as a cost-effective and accessible option for chronic low back pain self-management. Established in-person pain programs can provide the structure for an optimal mobile app adaptation. PainNavigator (PainNavigator, Inc) is an example of a mobile app that is based on an Ascension-Illinois group–based pain program—Pain Rehabilitation Outpatient-Camp.

**Objective:**

This was a prospective pilot clinical trial that evaluated the PainNavigator platform’s utility in low back pain management to inform future trial development.

**Methods:**

A total of 75 participants who used PainNavigator were studied. Pain, Enjoyment, and General Activity (PEG-3) scale scores and scores from a brief anxiety and depression scale based on the Patient Health Questionnaire-4 (PHQ-4) were obtained at baseline and following program completion. The PEG-3 total score was used, in addition to individual items—*Average Pain*, *Pain Effect on Enjoyment*, and *Pain Effect on Activity*. The PHQ-4 total score was also used, in addition to other individual items, including *Felt Depressed*, *Loss of Interest*, *Felt Anxious*, and *Difficult to Control Worry*. Paired sample *t* tests (2-tailed) compared mean differences in scores from before and after participants received the intervention.

**Results:**

The analysis found that PEG-3 (n=27) and PHQ-4 (n=27) total scores were significantly lower upon the completion of PainNavigator (*P*<.001 and *P*=.001, respectively). The findings showed a 36% reduction in PEG-3 total scores, a 40% reduction in pain intensity, and a 40% reduction in PHQ-4 total scores. Scores for individual PEG-3 scale and PHQ-4 items also significantly decreased. All PEG-3 measures had large effect sizes. The PHQ-4 total score and *Difficult to Control Worry* item had large effect sizes, while the other three measures had medium effect sizes.

**Conclusions:**

These findings show that PainNavigator has clinical significance in managing chronic low back pain and can be easily utilized to improve patient care. All PEG-3 scale and PHQ-4 measures significantly improved following the use of the platform, supporting the multidimensional, biopsychosocial approach to low back pain management. Differences in effect sizes may inform quality improvement investigations, such as optimizing features that impact measures with only medium effect sizes. This feasibility study demonstrates an effective protocol, and it will inform future, more extensive randomized controlled trials.

## Introduction

Chronic low back pain (LBP) is a multidisciplinary condition with significant socioeconomic implications. The Centers for Disease Control and Prevention has reported that approximately 20% of US adults experience chronic pain [1]. The economic impact of pain, when factoring in health care costs and productivity, has been estimated to be greater than that of heart disease, cancer, and diabetes [2]. LBP is a subset pain condition that also is the leading cause of years lost to disability [3]. LBP prevalence may also increase due to an aging population and can disproportionately burden low-middle–income people [4].

In addition to its profound impact on health care, LBP poses further challenges in management. LBP therapy begins with self-management, focusing on physical improvement and lifestyle modification; this can be scaled up to nonsurgical (including pharmacotherapy) and surgical interventions, as necessary [5]. In the past, medical management has emphasized these nonsurgical and surgical interventions. Although beneficial for the right individuals, this has potentially contributed to overusing imaging, opioids, and surgery, often complicating efficient patient care [6]. Additionally, external factors, such as insurance limitations, care provider reimbursements, and shifts in opioid prescribing practices, may affect a patient's treatment plan. More recently, there has been a focus on utilizing interdisciplinary therapies to treat LBP. This emphasizes a more holistic approach that focuses on the physical, psychological, and social impacts on an individual's life. This is known as the biopsychosocial management of chronic pain. Evidence of the benefits of this approach has been demonstrated across disciplines, with improvements seen in outcomes and significant cost savings [7,8]. However, most physicians lack sufficient training in biopsychosocial-based chronic pain treatment plans. Additionally, health care professionals' attitudes and beliefs about LBP have been linked to patients’ attitudes and treatment adherence, showing that effective treatment encompasses all involved [9]. Patients are often unable to manage concurrent visits and the costs of physical therapy, health psychology, health care providers, and therapists while also maintaining daily personal and professional responsibilities. Given the multidisciplinary nature of LBP and the socioeconomic implications of effective management, recent attention has been drawn to the importance of comparative effectiveness and randomized controlled trials for emerging LBP management strategies [5].

Mobile health (mHealth) apps may address this gap in LBP management. In addition to research demonstrating app efficacy, care providers and patients have gradually adopted such technology, which is likely a result of the accessibility of mHealth apps. Back pain apps have shown utility in delivering therapeutic pain interventions while bridging the gap between patients and care providers [10]. Apps have been developed to assess chronic LBP patients' thoracolumbar range of motion [11] and postural re-education [12]. Pain-centered mobile apps can also address postoperative pain assessment [13] and cancer pain management [14], demonstrating patient acceptance in ambulatory care. Meta-analyses that investigated the role of self-management and eHealth in LBP found sufficient evidence supporting their roles in pain and disability [15-17]. Du et al [16] further described that mobile platforms have advantages over web platforms with regard to their effect on pain and disability. Although many apps have been developed, differences among user interfaces may impact practicality, which may be addressed by focusing on user-centered designs [18]. LBP management has been evolving, and mobile apps appear to play an essential role in achieving lasting results, which is the goal of effective patient care.

An Ascension-Illinois group–based pain program—Pain Rehabilitation Outpatient-Camp—reported successful longitudinal results, including a 52% reduction in pain within 3 months, a 47% reduction in the risk of opioid misuse, a 40% reduction in pain disability, and a 60% reduction in depression. However, this in-person, 50-hour program (conducted over 6 weeks) was limited by accessibility and was correspondingly adapted into a mobile app interface—PainNavigator (PainNavigator, Inc). This self-guided program provides educational and movement-based modules to improve pain and function. PainNavigator's interdisciplinary approach and digital accessibility may exponentially increase cost savings for medical, insurance, and wellness groups. However, the platform's feasibility has not yet been assessed.

This research was a prospective pilot clinical trial that studied pain management questionnaire data from patients with chronic LBP before and after they used the PainNavigator software app. This study aimed to evaluate PainNavigator's utility by assessing pain scores and functional outcomes. We hypothesized that participants would experience positive outcomes, as depicted by improved subjective survey scores, after using the PainNavigator mobile app.

## Methods

### Study Design

This was a prospective pilot clinical trial that investigated the utility of the PainNavigator mobile app. This study assessed pain scores and functional outcome data from patients with chronic LBP before and after they used the platform. As this study was exploratory, all participants received the intervention, and there was no control group.

### Ethics Approval

Study participants were provided with a digital informed consent form through the mobile app, and they provided a signature on acceptance of the terms of consent. Participant data were deidentified for privacy and confidentiality protection. Compensation in the form of cash gift cards was provided for participation. This research was approved by the Ascension-Illinois Institutional Review Board (RIL20210036) and was carried out in accordance with the Helsinki Declaration's ethical standards.

### Participants

A total of 75 participants were recruited via health care providers within the Ascension-Illinois system. Participants either were given PainNavigator enrollment information, as deemed appropriate by their primary care provider during a clinic visit, or obtained the Ascension-Illinois flyer through Ascension-Illinois Marketing outlets. Participants were incrementally compensated up to US $80 in the form of cash gift cards based on the completion of modules. The inclusion criteria were men and women aged 18 years or older and those experiencing LBP for greater than 4 weeks that was nonsurgical and nonmalignant in etiology. Given the limited access among many people to multidisciplinary pain management clinics, this study aimed to target patients before they considered pain management clinics or those without access to such resources. The exclusion criteria were as follows: an age of under 18 years; the inability to complete the pain questionnaire in written English; the inability to utilize the software app; recent back surgery in the past 6 months; back pain due to malignancy; a diagnosis requiring surgery; uncontrolled depression, anxiety, or a severe mental disorder; severe medical conditions (including heart disease, lung disease, a history of strokes, and neurological disorders such as paralysis or uncontrolled seizures); patients who are advised against physical exercise or mental health self-therapy by a health care provider; patients undergoing interventional pain management techniques; patients undergoing any form of outpatient psychotherapy; pregnant patients; and adults who are unable to consent. Participant age and baseline pain duration demographics are shown in Table 1.

**Table 1:** Participant age and baseline pain duration demographics.

Variables		Participants who signed up for the program (n=75)		Participants who completed the program (n=30)	
		n	%	n	%
**Age range (years)**					
	18-29	7	9	0	0
	30-39	11	15	4	13
	40-49	13	17	7	23
	50-59	16	21	7	23
	≥60	28	37	12	40
**Pain duration**					
	1 to 3 months	6	8	1	3
	3 to 6 months	3	4	1	3
	6 months to 1 year	11	15	4	13
	1 to 3 years	12	16	5	17
	>3 years	43	57	19	63

### Protocol

**Figure 1:**
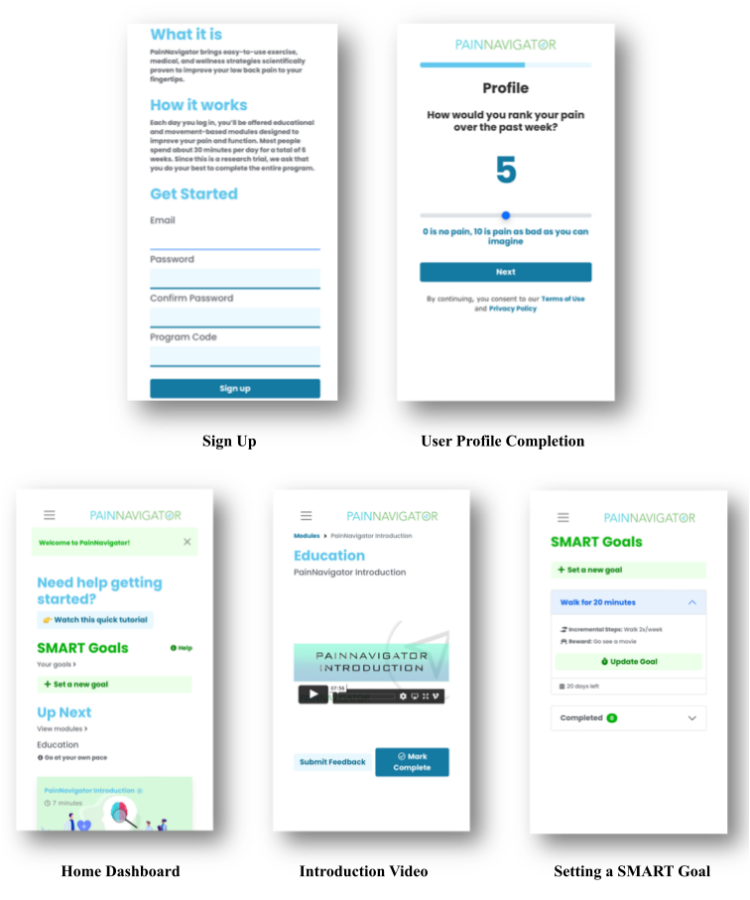
Screenshots of the PainNavigator app. SMART: Specific, Measurable, Attainable, Realistic, and Time-limited.

PainNavigator is a web-based mobile app that users can access for pain self-management (Figure 1). At baseline, the app gave participants pain and function questionnaires (Multimedia Appendix 1). These included demographics (ie, age, gender, and activity level); symptomatology (ie, the duration of LBP and the effect of LBP on behavior); treatments (ie, the names of opioids, the doses being taken [if applicable], and the likelihood of pursuing opioid or surgical intervention); the Pain, Enjoyment, and General Activity (PEG-3) scale; and the 4-item anxiety and depression questionnaire that was based on the Patient Health Questionnaire-4 (PHQ-4). This foundation promoted the development of a personal functional goal for users to achieve during the program. The app then guided users through prerecorded medical education and wellness strategy content, including evidence-based cognitive behavioral therapy techniques, yoga, mindfulness, and exercise therapy. The prerecorded content was led by a pain management physician, a health psychologist, a certified yoga instructor, and a doctor of physical therapy. The educational videos taught common causes of and solutions for pain and wellness strategies that users could learn for managing pain better. The movement modules taught exercises and stretches for back pain and mindfulness to emphasize a mind-body connection. Users set goals for activities that pain was holding them back from and, via weekly phone calls, worked one-on-one with a live wellness coach who supported goal achievement and program consistency. To solidify their understanding of the content, users completed postvideo actions, including the use of pain, mood, and food journals. Throughout the program, users leveraged these journals to identify triggers and drive behavior change. Upon finishing the program, users answered the completion survey, which included a pain and function questionnaire (Multimedia Appendix 2).

### Statistical Analysis

The data analysis included a comparison of deidentified, participant-inputted responses to questionnaires. Responses that were given before, during, and upon the completion of PainNavigator were analyzed for statistically significant changes in pain scores, as defined by standard statistical methods. Analyses were completed only for participants who answered each question, and composite scores, including the PHQ-4 and PEG-3 total scores, were only calculated for participants who answered each item that contributed to the scores. Data were analyzed in SPSS version 26 (IBM Corporation). The significance level was set to an *α* of .05. Data were first assessed for skewness. Paired sample *t* tests were used to compare mean differences in scores from before and after participants received the intervention. The Cohen *d* was calculated from the raw means that yielded effect sizes. Power was also calculated with G*Power version 3.1.9.7 (Heinrich-Heine-Universität Düsseldorf) [19] post hoc, using the sample size, *α*, and the effect size.

## Results

**Figure 2:**
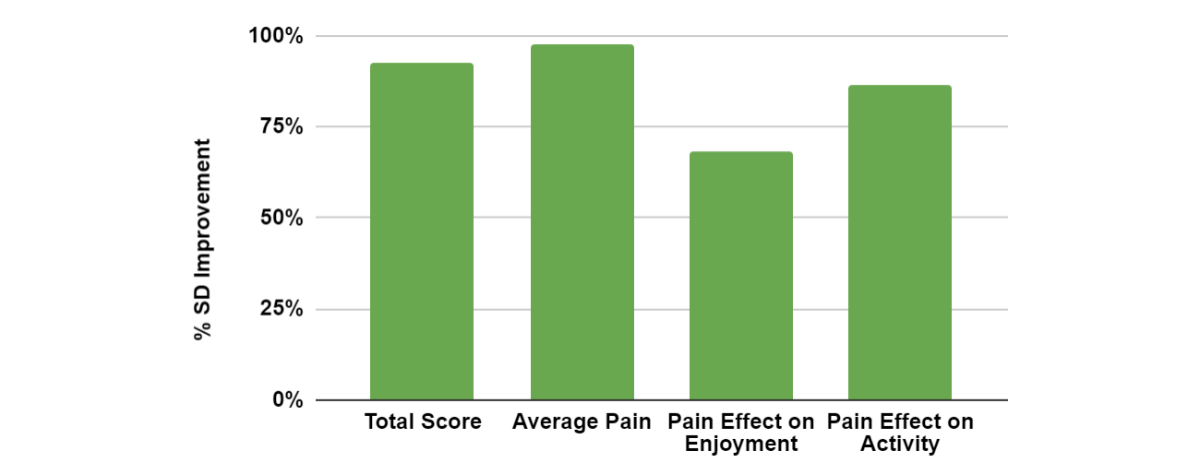
The percent SD changes from baseline to after the completion of PainNavigator for the Pain, Enjoyment, and General Activity scale (n=27). Effect size is denoted by color; medium effect sizes are yellow, and large effect sizes are green.

A total of 30 participants completed the program; however, 3 were excluded from analysis due to incomplete baseline PHQ-4 answers. Paired sample *t* tests were conducted for participants who completed all survey items before and after the intervention (n=27) to determine the effect of PainNavigator on pain, as measured by the PEG-3 scale, and its effect on anxiety and depression symptoms, as measured by the PHQ-4. The results indicated a significant difference in PEG-3 total scores from before (mean 16.703, SD 6.550) and after (mean 10.629, SD 5.204) participants completed PainNavigator (*t*_26_=7.639; *P*<.001). The 95% CI of the difference in means ranged from 4.439 to 7.708. Effect sizes (*d*) were also calculated by using the Cohen *d*. Cohen [20] suggested that a *d* of 0.2 is considered a small effect size, 0.5 represents a medium effect size, and 0.8 represents a large effect size. For the PEG-3 total score, a large effect size (*d*=1.027) was observed. Each of the three individual items—*Average Pain*, *Pain Effect on Enjoyment*, and *Pain Effect on Activity*—comprising the PEG-3 were also analyzed, suggesting significant differences and large effect sizes, as detailed in Table 2. The magnitude of change for each PEG-3 scale measure, which was standardized based on the baseline SD, is shown in Figure 2. Overall, on average, participants experienced pain decreases between baseline and the completion of PainNavigator.

**Table 2:** Pre- and postintervention measures for Pain, Enjoyment, and General Activity (PEG-3; n=27) and Patient Health Questionnaire-4 (PHQ-4; n=27) total scores and subscores. Paired *t* tests evaluated mean differences.

Variables			Baseline, score					End, score					Analysis		
			Mean		SD		Mean			SD		*P* value			Effect size, *d*
**PEG-3 items**															
	Total score	16.703		6.550		10.629			5.204		<.001^a^			1.027	
	Average Pain	5.482		2.230		3.310			1.605		<.001^a^			1.118	
	Pain Effect on Enjoyment	5.629		2.662		3.814			2.253		<.001^a^			0.736	
	Pain Effect on Activity	5.583		2.357		3.541			1.793		<.001^a^			0.975	
**PHQ-4 items**															
	Total score	5.814		3.101		3.481			3.309		.001^a^			0.728	
	Felt Depressed	0.963		0.979		0.555			0.751		.031^b^			0.468	
	Loss of Interest	1.333		1.000		0.814			1.001		.013^b^			0.519	
	Felt Anxious	1.814		0.962		1.148			1.099		.003^a^			0.645	
	Difficult to Control Worry	1.703		0.912		0.963			1.055		.002^a^			0.750	

^a^Significant at *P*<.01.

^b^Significant at *P*<.05.

**Figure 3:**
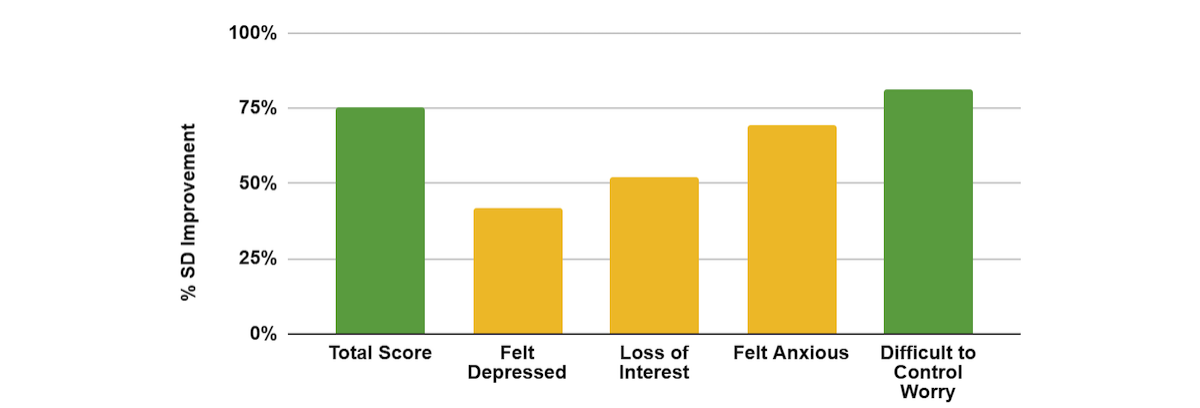
The percent SD changes from baseline to after the completion of PainNavigator for the Patient Health Questionnaire-4 (n=27). Effect size is denoted by color; medium effect sizes are yellow, and large effect sizes are green.

The results also indicated a significant difference in PHQ-4 total scores from before (mean 5.814, SD 3.101) and after (mean 3.481, SD 3.309) participants completed PainNavigator (*t*_26_=3.805; *P*=.001). The 95% CI of the difference in means ranged from 1.072 to 3.593. An effect size approaching large (*d*=0.728) was observed. Each of the four individual items from the PHQ-4—*Felt Depressed*, *Loss of Interest*, *Felt Anxious*, and *Difficult to Control Worry*—were also analyzed, suggesting significant mean differences with medium to large effect sizes between pre- and postintervention scores, as reported in Table 2. The magnitude of change for each PHQ-4 measure, which was standardized based on the baseline SD, is shown in Figure 3. Overall, participants observed decreases in anxiety and depression between baseline and the completion of PainNavigator. All metrics demonstrated significant decreases.

Moderately skewed results from the PHQ-4 at the end of the program (0.708) and the PEG-3 scale at the start of the program (0.627) were addressed by performing nonparametric tests to decrease the chances of making a type 1 error. Findings from related-samples Wilcoxon signed rank tests indicated that the null hypothesis would still be rejected for the PEG-3 scale (*P*<.001) and PHQ-4 (*P*=.002). Other data were also skewed and were analyzed the same way, and all data indicated that the decision to reject the null hypothesis appeared correct.

A post hoc power analysis was completed to determine achieved power. This was done with G*Power version 3.1.9.7 [19] for matched pair mean comparisons, using the sample size (n=27), *α* (.05), and the effect sizes of the PEG-3 total score (*d*=1.027) and PHQ-4 total score (*d*=0.728). Additionally, the highest (*d*=1.118) and lowest (*d*=0.468) calculated effect sizes were used to provide a range. For the PEG-3 total score *t* test, the power was calculated to be 0.99. For the PHQ-4 total score, the power was calculated to be 0.95. In the case of the largest effect size, the power was estimated to be 0.99. In the case of the smallest effect size, the power was estimated to be 0.64.

## Discussion

### Principal Findings

The preliminary results from this pilot trial support PainNavigator's initial acceptability and can help inform a larger randomized controlled clinical trial. Using the app significantly impacted all PEG-3 scale and PHQ-4 measures, indicating that the platform addressed essential components for the self-management of LBP. The findings show a 36% reduction in the PEG-3 total score, a 40% reduction in pain intensity, and a 40% reduction in the PHQ-4 total score. The magnitude of change for each measure illustrated these effects, with a positive percent change indicating score improvement (Figure 2 and Figure 3). The sample size was limited but acceptable for an initial pilot study. Given this sample size, the large effect sizes play an important role in study power. The strong power in this study indicates that the observed significant changes are likely clinically relevant and warrant a more rigorous study.

### Comparison With Prior Work

Published literature on pain-focused mHealth apps shows efficacy data that can be compared with data from PainNavigator. A meta-analysis of pain eHealth applications found that program durations of ≤8 weeks (“less intensive duration”) had an advantageous effect on reducing pain intensity [16]. The 8-week program described in this study similarly found significant improvements in pain, as assessed by the PEG-3 scale item *Average Pain*. These findings may suggest that concise programming facilitates user engagement and retention. Furthermore, the meta-analysis effect sizes of immediate and short-term pain intensity were found to be −0.16 (95% CI −0.30 to −0.02) and −0.27 (95% CI −0.43 to −0.11), respectively [16]. The absolute effect size for average pain following the PainNavigator intervention was 1.118, which is greatly higher in comparison. This may indicate that intrinsic app factors, such as design and functionality, assist with pain reduction.

Beyond pain measures, psychology may also play an important role in LBP management. An investigation of musculoskeletal pain rehabilitation outcomes found psychological and pain indices to be significantly intercorrelated [21]. This supports the biopsychosocial approach, as it appears to incorporate multidimensional patient care. PainNavigator demonstrated significant improvements in the psychological measures assessed by the PHQ-4. These findings may indicate the importance of psychological components in pain management programs.

As an emerging field, mHealth apps also have challenges. These include a general lack of quality mHealth app assessments [22] and the number of chronic pain apps that only offer 1 self-management strategy [23]. Many health apps are also mostly used until initial milestones, with significantly decreased usage afterward [24]. PainNavigator’s effect may be the result of the mobile app addressing some of the aforementioned challenges. PainNavigator's interdisciplinary approach introduces users to multiple strategies (eg, home exercise, yoga, wellness, nutrition education, etc) instead of focusing on only 1 modality. As such, users can utilize the modalities that provide the most benefits. Additionally, PainNavigator emphasizes function as an equal or greater part of the self-management of chronic pain when compared to pain scores. The wellness coach and personal goals hold users accountable for completing the program and provide a framework for continuing usage once initial milestones are met.

### Limitations

Due to the single-arm design and the lack of a control group, our results should be interpreted cautiously. Nevertheless, our results were quite promising for informing future rigorous tests of the PainNavigator intervention. Another limitation may be the usability of mHealth technology in older populations. Despite this, over half of the participants who completed this study were aged over 50 years (19/30, 63%). Other back pain mobile app studies additionally reported disability data, which this study did not explicitly assess. There were also differences between the number of enrolled participants and the number of those who completed all surveys, indicating a drop-off in responses. Lastly, financial compensations were given to participants for their participation in this study. Although this is a well-accepted practice in research studies, there is some debate about how this impacts compliance*.*

### Future Research

This study’s findings will guide future research into pain-centered mHealth app efficacy and scalability. For example, the analysis found that the *Felt Anxious*, *Felt Depressed*, and *Loss of Interest* PHQ-4 submeasures had medium effect sizes, whereas large effect sizes were found in all other measures; a future focus may be directed on these targets. Since user engagement with mHealth apps is essential to their efficacy, many studies have prioritized users’ needs during quality improvement and described their methodology [25,26]. Clinician feedback also may help direct LBP mobile app quality improvement [27]. Accounting for both types of input is essential, given the need for dynamic communication between patients and physicians. This feasibility study demonstrates that our protocol works, and more extensive randomized controlled trials will be conducted. mHealth app development is a dynamic process centered around providing patients with the best care.

### Conclusion

The PainNavigator mHealth app showed LBP management utility in this initial pilot trial. The significant improvements in all PEG-3 scale and PHQ-4 measures illustrate potential multidimensional, biopsychosocial management that is easily accessible to patients. This platform demonstrates clinical significance and can be easily utilized to improve patient care. Further randomized controlled trials are needed to expand upon these initial findings and explore the functional role of the PainNavigator platform in clinical settings.

## Appendix

Multimedia Appendix 1 Initial user questionnaire.

Multimedia Appendix 2 Completion questionnaire.

## Abbreviations

LBP: low back pain
mHealth: mobile health
PEG-3: Pain, Enjoyment, and General Activity
PHQ-4: Patient Health Questionnaire-4
