# Supplementary figures and images for: An Evaluation of a Mobile App for Chronic Low Back Pain Management: Prospective Pilot Study
Source: JMIR Form Res. 2022 Oct 13;6(10):e40869. doi: 10.2196/40869 (PMC9614628; doi:10.2196/40869)

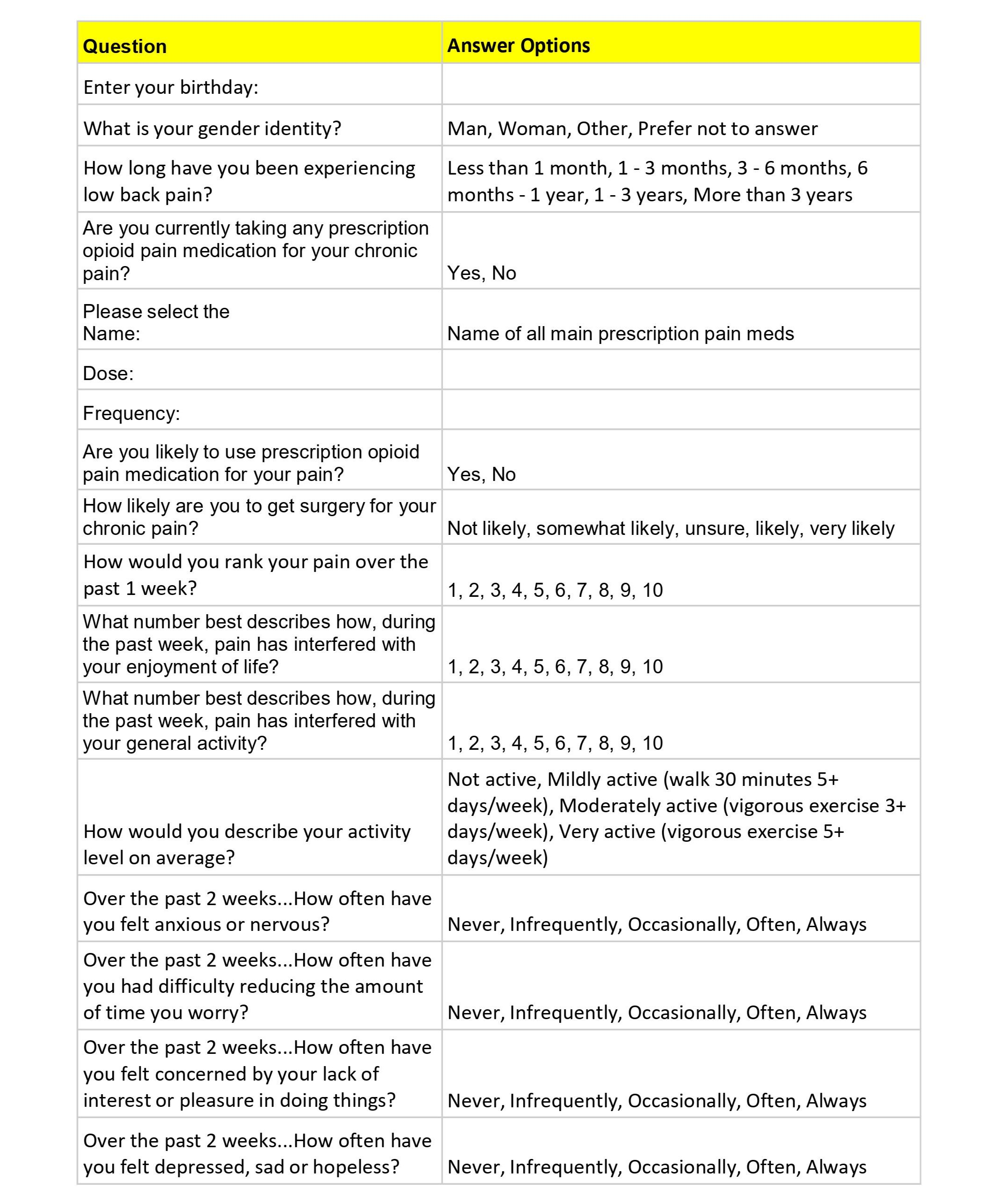

Supplement: Multimedia Appendix 1 [file formative_v6i10e40869_app1.png]

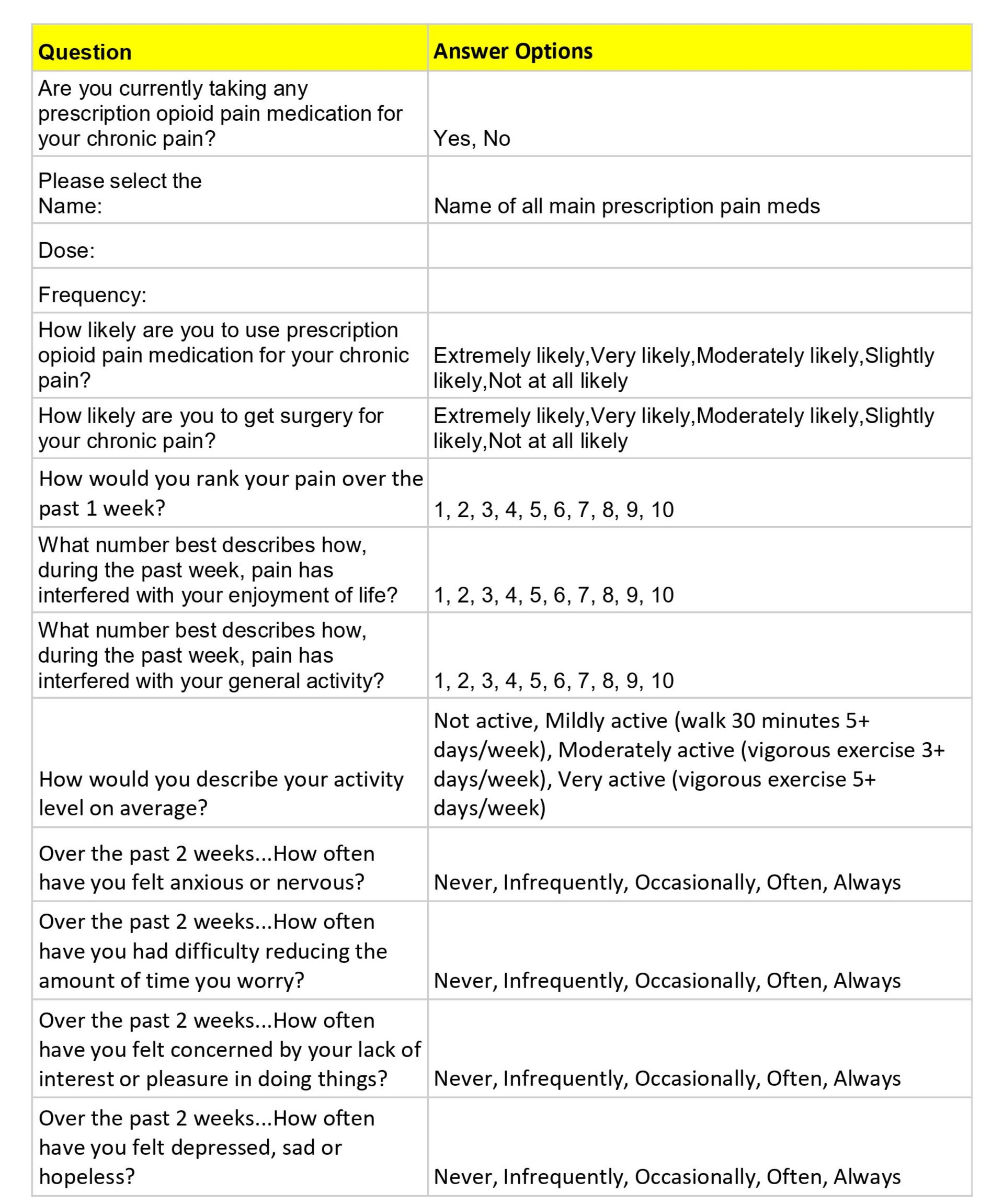

Supplement: Multimedia Appendix 2 [file formative_v6i10e40869_app2.png]
